# Children with Autism Spectrum Disorder and Patterns of Participation in Daily Physical and Play Activities

**DOI:** 10.1155/2015/531906

**Published:** 2015-06-15

**Authors:** Amir Hossein Memari, Nekoo Panahi, Elaheh Ranjbar, Pouria Moshayedi, Masih Shafiei, Ramin Kordi, Vahid Ziaee

**Affiliations:** ^1^Neuroscience Institute, Sports Medicine Research Center, Tehran University of Medical Sciences, Tehran, Iran; ^2^Department of Neurology, University of California, Los Angeles, CA, USA; ^3^Growth and Development Research Center, Tehran University of Medical Sciences, Tehran, Iran

## Abstract

Autism spectrum disorder (ASD) indicates several neurodevelopmental impairments which may end in impairments in motor or physical activities. Daily physical activity involvement was investigated in a total of 83 children (52 boys and 31 girls) with ASD aged 6–15 years. Results indicated that only 10 (12%) of children with ASD were physically active. Children were predominantly engaged in solitary play rather than social play activities. Gender, family income, and household structure were found to be associated with activity scores. Financial burden and lack of opportunities were noted as the leading barriers to physical activities. In conclusion, findings indicated a low rate of physical activity participation in children with ASD that is closely associated with sociodemographic variables.

## 1. Introduction

Autism spectrum disorders (ASDs) describe a group of neurodevelopmental conditions in which the individuals face challenges with social engagement and age-appropriate play and fail to develop appropriate peer relationships according to their developmental level [1]. Although young people are frequently recommended to participate in leisure activities including play, sports, hobbies, and social activities, children with ASD tend to spend time in passive play and maladaptive behaviors and they are less likely to spontaneously participate in organized leisure activities such as sports [2, 3].

It could be attributed to their significant deficits in development of motor development and physical activity (PA) behavior [4, 5]. Social and behavioral impairments in ASD can limit children opportunity to participate in physical activity and recreation programs that eventually end to their inactivity [6]. Physical inactivity predisposes children with ASD to several comorbid conditions such as overweight and obesity [7, 8]. To assess key correlates of physical activity, previous studies frequently addressed social variables as critical factors contribute to ASD children physical activity [9]. For example, Pan [10] showed that children with ASD who had lower social engagement with adults displayed lower levels of physical activity than children had higher social involvement. Indeed although children with ASD receive rehabilitation services from an early age in order to improve daily performance and enhancement of active life, PA and leisure aspects of quality of life (QOL) are underestimated in children with ASD and their families [11, 12]. To address the children needs, parents and caregivers have to spend many resources while making a balance between children needs and those of family or guardians is a difficult task. Thus recently, studies examining QOL in a wide range of individuals with ASD indicated that adults with ASD have lower scores in wellbeing measures [13, 14], and children also show a subideal outcome [15, 16]. A recent study on ASD demonstrated a positive connection between cheerfulness and participation in a quality leisure program; authors also indicated that satisfaction is also correlated with leisure activities in individuals with ASD [17].

Although some studies could not show any differences between the physical activity levels of children with and without ASD, there is a general consensus that children with ASD did not participate in enough PA necessary to meet the activity guidelines for wellbeing [9]. Most recent review of literature confirmed that children with ASD fall short of the recommended physical activity levels and experience challenges in physical activity and physical education settings and thus recommended strategies to improve the physical activity statistics and quality of life among children with ASD [18]. Limited research, to date, has looked at the barriers and facilitators of PA among children with ASD [19]. However, a number of barriers to physical activity from individual to social and environmental make PA participation of children with ASD more difficult and may result in increasing their sedentary activities. A rare study examining parent-reported barriers to PA in children with ASD reported significantly greater amount of barriers compared to TD children. Barriers reported by parents are too much supervision needed compared to TD children, lack of skills, few friends, and exclusion by other children which are the most important barriers [20]. However, children with ASD themselves rated interpersonal (such as screen activities), physical (such as lack or unsafe equipment), and community (such as lack of transportation to physical activity programs) factors as the most frequent barriers [19].

On the other hand, there are facilitators from personal (individual versus team activities) to collective (such as social support) that help children with ASD to involve in a PA program. Particularly the association between PA and social support has been established among children with ASD [19]. However a multiaspect approach is needed to assess the PA and leisure participation of children and adolescents with ASD.

Of relevance to current study, it is important to examine whether the children with ASD are provided with enough opportunities to participate in physical activities and what factors are playing a role in their physical activities. Furthermore, identifying contributing factors into PA will be imperative to enhance the efficacy of interventions aimed to improve active life/wellbeing of children with ASD. Therefore, we aimed to assess the participation of a school based sample with ASD in physical and daily activities. We further sought to examine individual (e.g., age and clinical symptoms) and social (e.g., household structures) factors contributing to the level of participation in leisure physical activities.

## 2. Methods

### 2.1. Participants

A total sample of 83 children (53 boys and 31 girls) with high functioning ASD (IQ > 70) aged 6 to 15 years (Mean = 9.8, SD = 1.8) were recruited from four autism specific schools in Tehran. All of the subjects had received a clinical diagnosis of ASD (autism, Asperger's, or pervasive developmental disorder, not otherwise specified) by a child psychiatrist or clinical psychologist and the diagnosis was confirmed using the revised Autism Diagnostic Interview (ADI-R) [21]. This cross-sectional study was approved by the Medical Ethics Committee of the Tehran University of Medical Sciences. Parents or caregivers of children provided informed consent prior to participation.

### 2.2. Measures

Physical activity involvement during leisure time was examined using a modified checklist adapted from Godin-Shephard Leisure Time Questionnaire (GLTEQ). We aimed to evaluate activities (at least 15 minutes) over a 7-day period asking two questions. The first question addresses the intensity of physical activities: strenuous (e.g., running, football), moderate (e.g., easy bicycling, easy swimming), and mild (e.g., yoga, easy walking). Since children with ASD were not acquainted to complete a self-report questionnaire, we modified the questions in order for parents/caregivers to reply. For instance “how many times on the average do you do the following kinds of…?” was replaced by “how many times on the average does your child do the following kinds of exercise…?” We asked parents to consider physical activities all day long (including school time) when they answered the questions. They attended the schools frequently and closely observed the children activities. Also each child's teacher was asked to help parent to include school time physical activities when asking the questions on “how many times on the average does your child do the following kinds of exercise…?”

Finally a composite score was calculated as Activity Score = (9 × (number of strenuous exercise episodes)) + (5 × (number of moderate exercise episodes)) + (3 × (number of mild exercise episodes)) [22]. An additional question was presented as “During a typical 7-Day period (a week) how often does your child engage in any regular physical activity long enough to work up a sweat (heart beats rapidly)?” with three answer options as “Often”, “Sometimes,” and “Never/Rarely.” An overall total high score on both questions reflects a high level of physical activities. Prior research showed an acceptable criterion validity and also reliability scores (0.74 and 0.80) [23]; our data on a subsample of participants (25 parents) also showed a good test-retest reliability scores (0.79 and 0.81). According to the activity guidelines, children were supposed to participate in exercise activities at least 60 minutes at a moderate to vigorous intensity on most (five) days of the week in order to be considered “active” (GLTEQ score = 5 × 5 days × 4 (60/15 min) ≥ 100) or they are considered “inactive” if their activity score was lower than the minimum recommendations (GLTEQ score < 100).

To assess the barriers to PA, parents were asked to specify the most frequent barriers to participation in leisure physical activity of their child. A list of barriers was provided including expense, lack of resources/opportunities, time limitation, motivation, and fear of injury and also an open item as “other barriers.” Furthermore, parents were asked to complete a daily activity logbook for children, which was designed to obtain information about each child on their hourly engagement during a typical day [24]. Parents rated how much time, in average, children spent on solitary, social, home teaching, TV, feeding, school, and also sleep on daily basis. Among those daily activities, social play (i.e., time spent on playing with peers) and solitary play activities were used for current study. This questionnaire showed a good content validity and a satisfactory test-retest reliability (intraclass correlation = 0.69, *p* < 0.001).

Moreover, we administered the Autism Treatment Evaluation Checklist (ATEC) to parents/caregivers to monitor the severity of autism symptoms. ATEC is a valid and helpful instrument to evaluate the severity ASD symptoms in children with ASD [25, 26]. The checklist has four subscales, including language, sociability, sensory/cognitive awareness, health/physical/behavior, and the total score (overall severity).

Finally, background demographic information of the participants was reviewed by the first author interviewing the families and using their medical profiles. In the next step, parental demographic variables including household structure (single parent versus two parents' family), household income, and the highest level of education obtained by parents were also recorded. Parental education was examined by one question that asked participants to report the highest degree completed by either the father or the mother. For the current study, three education categories were created including low (diploma and below), medium (bachelor and below), and high (master and above). Participants were also asked to report total household income. For use in this paper, household income was categorized into four groups using the poverty income ratio (based on poverty threshold from national central bank report). These categories ranged from below the poverty limit to incomes greater than three times the poverty limit.

### 2.3. Data Analysis

Descriptive data for general records were reported (Mean ± SD). Independent *t*-test was conducted to evaluate the statistical significance for the observed differences across gender (boys and girls) in outcome measurements (physical activity score or daily activity measures). Furthermore, in order to compare the time spent on solitary play and social play in total studied population, a paired *t*-test analysis was conducted. Association between leisure scores or daily activities time and parental and child factors were assessed by correlation analysis. The significance level was set at 0.05 to consider an outcome meaningful. Analyses were performed using the statistical package for the social sciences (SPSS) software version 17 for Windows (SPSS Inc., Chicago, IL, USA).

## 3. Results

The descriptive characteristics of children and their families are shown in Table 1. Children with median age of 9.5 (8.5–11.3) were assigned to this study. Eighty-nine percent of children had no or only one sibling. From all children, 21 (25.3%) lived in single-parent families. Composite score for leisure time activity was in average 47.7 and SD = 19.3. However, it was very striking to find that only 10 (12%) of children with ASD were active and 73 (88%) were inactive based upon activity guidelines and activity score measured by the GLTEQ. Addressing the frequency of activity participation, results showed that only 6% of children with ASD “often” participated in physical activities, whereas 85.5% of them had “never/rarely” participated and 8.5% were “sometimes” involved in physical activities. Furthermore, *t*-test analysis of GLTEQ composite score showed that boys with ASD participated in physical activities (58.8 ± 22.1) more than girls with ASD (35.5 ± 14.5) (*t* = 4.31, 95% CI: 12.48–33.13, *p* < 0.001). Examining the correlates of children physical activities revealed that expectedly older children were less active than younger children (*r* = −0.298, *p* = 0.003).

There was no association between severity of disorder or parental education level and the activity score, but physical activity participation was positively correlated with the poverty income ratio (*r* = 0.31, *p* = 0.005). *t*-test analysis showed that children in single-parent families had significantly lower activity scores than children in two parent's families (*t* = 3.91, 95% CI: 9.31–29.64, *p* < 0.001).

Table 2 shows the measurements obtained from daily activity logbook. Based on the results obtained from independent *t*-test, girls were more involved in solitary play compared to boys (*t* = 3.626, 95% CI: 31.01–106.22, *p* < 0.001). The results of paired *t*-test showed that children were predominantly more engaged in solitary play compared with social play activities (*t* = 9.41, 95% CI: 65.68–100.80, *p* < 0.001). Correlation analysis between daily activities and symptoms severity scores showed that social play participation was negatively correlated with language impairment (*r* = −0.25, *p* = 0.016), sensory/cognitive awareness deficit (*r* = −0.26, *p* = 0.013), and also overall severity score (*r* = −0.29, *p* = 0.005).

Finally, parents reported that leading barriers of children adherence to physical activities were “Expense” (31.7%) and “Lack of Resources and Opportunities” (30.1%) followed by “Time” (19.5%), “Motivation” (17.1%), and “Fear of Injury” (1.2%).

## 4. Discussion

Daily physical and play activities have an important role in the psychosocial development of children. In fact an appropriate activity profile prevents them from isolation in adulthood [27] and significantly influences the wellbeing of children [17, 28]. Nevertheless, there was lack of studies assessing daily activities participation in children with ASD and investigating the impact of individual and environmental factors on their physical activity parameters.

Results from the current study indicates that most of the children with ASD do not have adequate physical activity participation since only few of our children met the minimum physical activity criteria. Several studies have discovered that individuals with disabilities are more likely to be inactive and due to abundance of impediments they are less likely to participate in activities when they are compared with the general population [29–32]. Their findings show that ASD and the severity of intellectual impairments place the people with disabilities at a higher risk for sedentariness [33, 34]. A number of factors can limit the participation of children with ASD in daily physical activities. Those mainly include lack of positive experiences in exercises, frequent failures, emotional impairments, and low self-esteem [34].

However, our data showed that such low participation was mostly due to the financial complaints and lack of resources or opportunities as reported by the parents. Moreover, there were other factors (e.g., time constraints, lack of motivation, and fear of injury) which can further limit the participation of autistic children in activities. Interestingly, data from another developing country revealed similar barriers such as financial complaints, lack of knowledge, and perception of the situation in an ASD sample [35]. Although there are differences in measurement of barriers across previous studies, almost similar patterns of barriers including time limits and financial constraints were reported as leading barriers to activity participation in children with disabilities particularly ASD [15, 36, 37]. Indeed this finding is not limited to ASD and previous data from individuals with other disabilities revealed that disabled people face a number of barriers to PA participation even more than healthy population. Expenditures of the child's medical care impose a financial burden on families with an autistic child and therefore they require more financial resources. They also have to limit their productive working times in order to take care of their difficult children, which in turn will further challenge the possibility of secure a financial resource expansion [38].

One of the important findings of the present study is that children from low-income families show lower level of PA than children from high-income families. Indeed family income is a determinant of health behavior. Children who grow up in a low-income family are more likely to live a sedentary lifestyle and experience more health problems related to physical inactivity compared to children from higher income families [39]. There are a number of physical and social barriers to physical activity for low-income families including low access to parks and recreational services, traffic conditions and air pollution, lack of relevant alternatives of transportation, and lack of enough social support for physical activity. On the other hand, low-income families are often less able to overcome these barriers [40]. Due to financial constraints, there are fewer alternatives available for low-income people; for example, they are not able to spend on a health club or recreation center membership [41]. It can be expected that the problem is more complicated in families with an ASD child. Thus, the economically disadvantaged ASD families may show a lower preference to participate in physical activities [42]. Furthermore, some parents have increasing concerns about their child's health and possibility of an injury, which can explain their lack of interest toward activity participation of their autistic child.

The household structure has been identified as another independent correlate of activity participation. Single parents experience a number of work-related or housing problems. Furthermore, they report lack of time and financial resources as the main impediments to participation in activities [32]. Our findings provide additional evidence regarding the effect of household structure on the leisure time activity involvement in children with ASD. However, it is not clear if other variables such as presence of a sibling may influence the opportunities to engage in social play and daily social activities inside the family environment.

Expectedly children with ASD showed a remarkably low social but high solitary play activity during a typical day. Indeed this finding may reflect the characteristic of autism itself. A previous research has shown that the characteristics of ASD as social, communicative, and motor impairments increase the likelihood of loneliness and decrease the opportunities for interactions in individuals with ASD [32]. Previous studies suggested that a lower level of social play activities in addition to autistic character difficulties can have serious developmental and social consequences [43–45]. Examining the apparent role of autism symptom severity, we observed that the children with greater deficits (e.g., in communication) had a lower engagement in social play activities. These results are in line with previous studies which indicated that there is an inverse correlation between severity of communication impairment and the level of life participation in individuals with disabilities. In fact, previous studies indicated that individuals with more severe motor or physical impairments or cognitive disabilities are at a higher risk of being excluded from daily activities [46, 47].

Our findings also indicated that there is a significant age and gender difference in level of physical activity and this is in line with studies of ASD and general population. Expectedly we documented the negative effect of age on PA in children with ASD. It can be explained that older children have low opportunities to participate in physical and recreation activities. Furthermore, age may decrease the children motivation to participate in complex motor or physical activities.

We also indicated that gender (in favor of males) influences the daily physical and play activities of children. Gender differences in ASD characteristics revealed that males with ASD show more stereotypic and repetitive behaviors while there are more communication deficits in female counterparts [48]. In addition, there is more achievements in motor skills and social competence in boys than girls with ASD [49]. One can argue that being a girl is associated with poor outcome in physical activity participation in ASD.

### 4.1. Limitations

Several limitations of this study have to be acknowledged. First, the cross-sectional design prevents an understanding of the exact nature of the daily activity participation, particularly with respect to its determinants. Second, while a neurotypical control group was not included, it would be helpful to compare the scores of physical activity participation between neurotypical controls and children with ASD. Third, the measures solely relied on the parents' (or teachers) self-report information; thus, recall bias maybe a potential limitation.

### 4.2. Conclusion

In conclusion, only a small part of children with ASD are physically active according to activity guideline. Financial concerns, lack of opportunities, and sociodemographic factors are indicated as major limitations of their physical activities.

## Figures and Tables

**Table 1:** General information of the children with ASD.

Total (*n* = 83)	Frequency	Percent
Child sex		
Boys	52	63.7
Girls	31	37.3
Number of siblings		
No sibling	32	38.5
1	42	50.6
2	6	7.2
3	2	2.4
4	1	1.2
Parental education level		
Low (diploma and below)	40	48.1
Medium (bachelor and below)	18	21.7
High (Master and above)	25	30.2
Family poverty income ratio		
<100%	8	9.6
100–200%	31	37.3
200–300%	23	27.7
>300%	21	25.3
Household structure		
Single parent	21	25.3
Two parents	62	74.7

**Table 2:** Time spent in social or solitary play activities (Minutes) in children with ASD by gender differences.

	Overall	Boys	Girls	*p* value^*∗*^
Solitary play (min/day)	94.0 ± 81.1	78.4 ± 72.9	147.0 ± 86.8	<0.001
Social play (min/day)	11.9 ± 28.2	11.8 ± 27.2	11.9 ± 31.9	0.99

^*∗*^Independent *t*-test analysis between boys and girls.
